# Effect of medical treatments in disseminated peritoneal leiomyomatosis: a case report

**DOI:** 10.1093/jscr/rjac166

**Published:** 2022-06-10

**Authors:** Anne-Sophie Navarro, Martina Aida Angeles, Claire Illac, Bérénice Boulet, Gwenael Ferron, Alejandra Martinez

**Affiliations:** Department of Surgical Oncology, Institut Claudius Regaud–Institut Universitaire du Cancer de Toulouse (IUCT), Oncopole, Toulouse, France; Department of Surgical Oncology, Institut Claudius Regaud–Institut Universitaire du Cancer de Toulouse (IUCT), Oncopole, Toulouse, France; Department of Pathology, Institut Claudius Regaud–Institut Universitaire du Cancer de Toulouse (IUCT), Oncopole, Toulouse, France; Department of Radiology, Institut Claudius Regaud–Institut Universitaire du Cancer de Toulouse (IUCT), Oncopole, Toulouse, France; Department of Pathology, Institut Claudius Regaud–Institut Universitaire du Cancer de Toulouse (IUCT), Oncopole, Toulouse, France; Department of Pathology, Institut Claudius Regaud–Institut Universitaire du Cancer de Toulouse (IUCT), Oncopole, Toulouse, France

## Abstract

Disseminated peritoneal leiomyomatosis (DPL) is a rare gynecologic disease involving multifocal proliferation of myomas. The pathogenesis remains unclear. Although there is no standard treatment, medical therapies have attempted to suppress estrogen levels by using gonadotrophin-releasing hormone agonist and aromatase inhibitor (AI) therapy with differing degrees of success. Surgery is also an option in symptomatic patients, and in the event of partial or no response to medical treatments. We report a case of DPL in a young woman with a previous history of myomectomy. She was treated sequentially with ulipristal acetate and AI.

## INTRODUCTION

Disseminated peritoneal leiomyomatosis (DPL) is a rare gynecologic disease involving multifocal proliferation of myomas that mainly affects women of reproductive age. The pathogenesis remains unclear, although three main theories have been proposed: hormonal, genetic and iatrogenic after laparoscopic myoma morcellation [1–4]. High levels of female gonadal steroids seem to play an important role in the pathogenesis of the disease [5]. Histopathologic analysis reveals findings similar to common uterine leiomyoma: typical smooth cell muscle proliferation and positive staining for gonadal hormone receptors.

Although there is no standard treatment, medical therapies have attempted to suppress estrogen levels by using gonadotrophin-releasing hormone agonist (GnRH) and aromatase inhibitor (AI) therapy with differing degrees of success. Surgery is also an option in symptomatic patients, and in the event of partial or no response to medical treatments. More recently, a selective progesterone receptor modulator, ulipristal acetate, which is already used to treat uterine myomas, has been used for this medical condition.

We report the case of a young patient desiring pregnancy who presented a DPL. She was treated sequentially with ulipristal acetate and AI and had a partial response.

## CASE REPORT

This 34-year-old woman had a history of pedicle myoma operated by laparoscopy 7 years prior to present symptoms. She did not have any previous history of cancer or other relevant medical condition. During the first surgery, the patient underwent a laparoscopic myomectomy of a 10-cm pedicle myoma localized in the right uterine horn. The tumor was extracted by morcellation without endobag protection. Histopathologic diagnosis confirmed the benign nature of the uterine leiomyoma.

**Figure 1:**
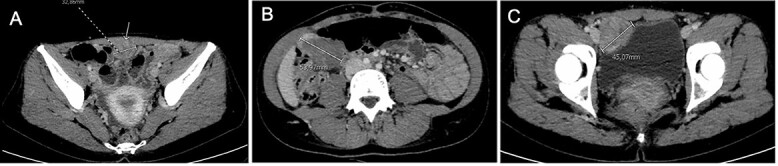
Computed tomography at diagnosis showing the three tissular lesions adherent to the anterior abdominal wall. (**A**) Tumor in contact with infra-umbilical linea alba. (**B**) Lesion located at the right hypochondria. (**C**) Tumor situated in the right iliac fossa near the bladder.

She presented to the emergency department with acute abdominal pain in February 2017. Thoraco–abdomino–pelvic computed tomography (CT) revealed three tissular tumors without evidence of calcification or fat signal adherent to the anterior abdominal wall: one of 34 mm in contact with the infra-umbilical linea alba, another of 44 mm in the right iliac fossa near the bladder and a third of 47 mm in the right hypochondria in contact with the right colic angle (Fig. 1). A transabdominal ultrasound-guided biopsy of the infra-umbilical tumor revealed fusiform cellular proliferation corresponding to smooth muscle-like cells. Based on these findings, the diagnosis of DPL was suspected, and the patient was referred to our center.

**Figure 2:**
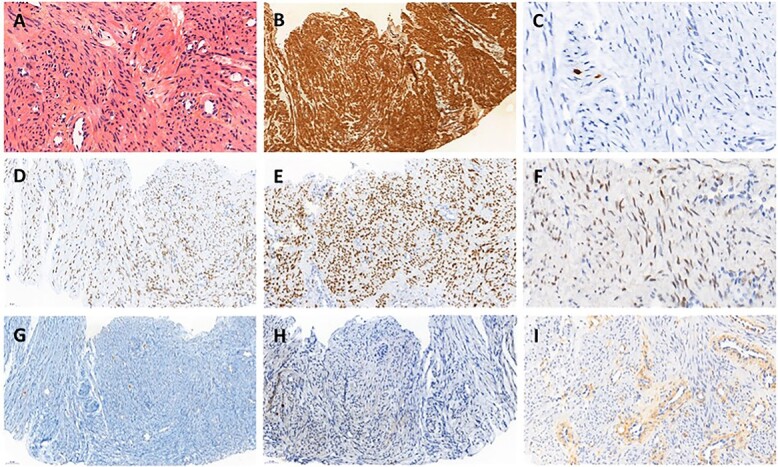
Histopathology of biopsy. (**A**) Smooth muscle cells with bland cytology, hematoxylin eosin stain ×400. (**B**) Strong positivity for smooth muscle actin, ×200. (**C**) Proliferative index evaluated with Ki67 was very low, ×200. (**D**) Strong and diffuse nuclear expression of estrogen receptors, ×200. (**E**) Strong and diffuse nuclear expression of progesterone receptors, ×200. (**F**) Heterogeneous expression of androgen receptors, ×200. (**G**) Tumor cell negativity for CD117, ×200. (**H**) Tumor cell negativity for HMB45, ×200. (**I**) Tumor cell negativity for EGFR, ×200.

A histopathological review of the slides was performed by an expert gynecologic oncology pathologist confirming the diagnosis (Fig. 2). Immunochemistry showed the tumor cells were positive with smooth muscle actin, desmin, caldesmone and hormonal receptor staining as described in leiomyomas [6].

**Figure 3:**
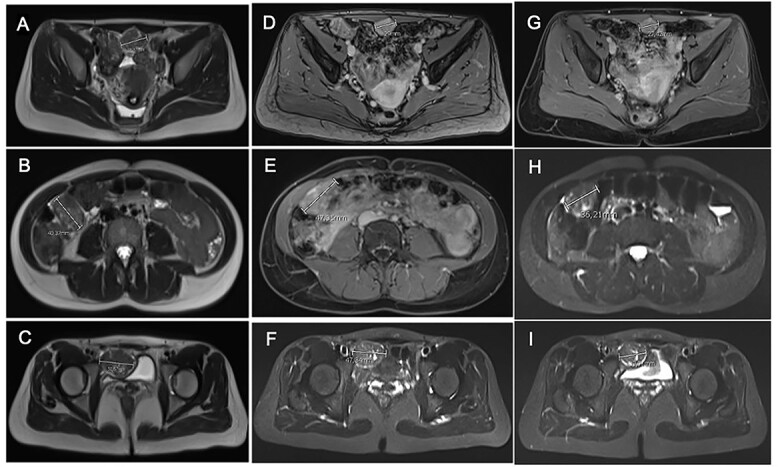
Comparative magnetic resonance imaging (MRI). (**A**–**C**) MRI after 3 months of acetate ulipristal. (**A**) Tumor in contact with infra-umbilical linea alba; (**B**) lesion located in right hypochondria; (**C**) tumor situated in right iliac fossa near bladder. (**D**–**F**) MRI after 3 months of AI and GnRH agonist. (**G**–**I**) preoperative MRI.

After tumor board discussion, hormonotherapy was recommended. One month after starting Letrozole, the patient expressed her desire for pregnancy and the treatment was switched to ulipristal acetate 5 mg per day. After 3 months of treatment, the abdominal pain persisted without any improvement and MRI re-evaluation showed stability compared with the previous CT (Fig. 3A–C). Given the inefficacy of this treatment, letrozole 5 mg per day was resumed in association with a Gonadotrophin-releasing hormone (GnRH) agonist (Triptorelin 12.5-mg intramuscular 1/month). After 3 months of treatment, MRI revealed a 10% reduction in the size of the three nodules (Fig. 3D–F). Abdominal pain was partially improved and localized in the right hypochondrium. The tumor board proposed MRI reevaluation after 6 months of effective treatment and surgical excision if the symptoms persisted.

**Figure 4:**
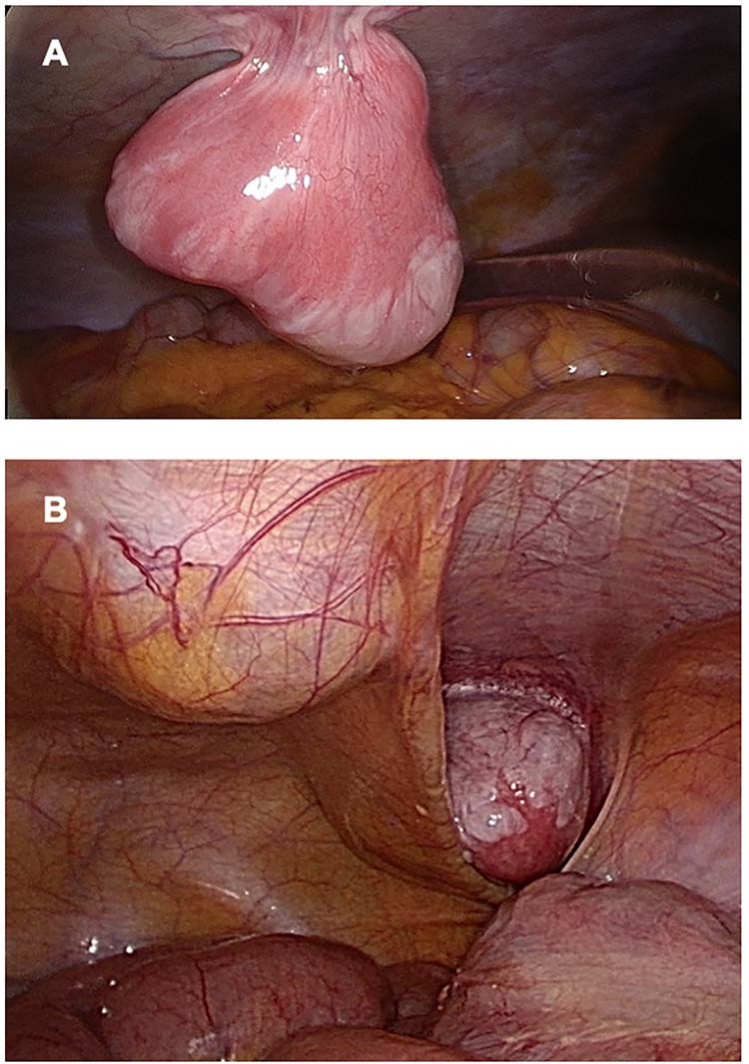
Three lesions seen by laparoscopy. (**A**) Lesion located in right hypochondria; (**B**) Right iliac fossa and linea alba tumors.

**Figure 5:**
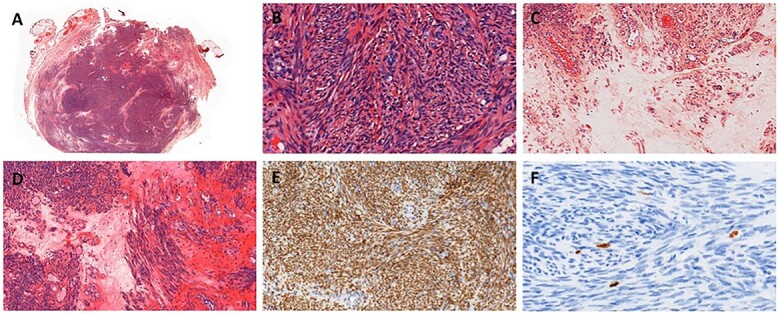
Histopathology of resection. (**A**) Hematoxylin eosin stain. (**B**) Spindle-shaped cells without atypia, hematoxylin eosin stain ×400. (**C**) Edema, hematoxylin eosin stain ×200. (**D**) Edema and fibrosis, hematoxylin eosin stain ×200. (**E**) Nuclear strong and diffuse expression of estrogen receptors, ×200. (**F**) Proliferative index evaluated with Ki67 ~1%, ×400.

Treatment was pursued for another 3-month cycle with persistent abdominal discomfort. Control MRI showed the persistent benefit and stability of the three lesions (Fig. 3G–I). Laparoscopy was performed after a total of 6 months of AI and GnRH agonist. Inspection of the abdominopelvic cavity revealed three tumors located in the right hypochondrium, the left para-vesical fossa and the median trocar scar infiltrating the abdominal wall. All three lesions were excised (Fig. 4). Definitive histopathological results showed spindle-cell proliferation of smooth muscle cells (Fig. 5). There was no nuclear atypia, necrosis or mitosis, but the proliferation seemed modified with edema and fibrosis, probably due to the treatment. Tumor cells were smooth muscle actin-, desmin- and caldesmone-positive, confirming their smooth muscle nature. They were CD117- and HMB45-. Ki67 was close to 1%.

No complications were observed in the postoperative course. After confirmation of the benign histological condition, no adjuvant therapy was offered to the patient. After 4 years of follow-up, she is disease-free.

## DISCUSSION

DPL is a rare gynecologic disease described for the first time in 1952 [7]. The development of multiple myomas in the abdominal cavity could be explained by the theory of Larrain *et al*. whereby fibroid fragments left behind after morcellation are parasitized by the blood vessels of the peritoneum and mesentery [4].

Medical treatments to date have sought to reduce estrogen levels. As DPL expresses progesterone receptors, ulipristal acetate seems to be an alternative treatment for young women. It has been used to treat symptomatic uterine leiomyoma as it reduces the tumor volume, pain and blood loss. Two randomized controlled trials found no difference between ulipristal acetate and leuprolide acetate in controlling uterine bleeding [8, 9]. However, since March 2020, it has been suspended by the European Medicines Agency due to hepatotoxicity reported in several patients [10]. In our case, it was used long before any side-effects were evidenced. Benlolo *et al*. reported a case of a 23-year-old woman without any history of myoma who underwent a 3-month trial of ulipristal acetate (5 mg per day) [11]. She presented a history of left lower quadrant abdominal pain. Thoraco–abdomino–pelvic CT revealed innumerable small, hypervascular soft tissue nodules throughout the abdomen, omentum and pelvis. She received five treatment cycles and had an excellent symptomatic and radiologic response. Another case of ulipristal acetate prescription in this indication was reported by Verguts *et al.*, patient was asymptomatic after 12 months of treatment [12]. Our patient did not report any clinical improvement after a treatment cycle with ulipristal acetate, even though MRI showed tumor stability.

AI is commonly used to treat breast cancer. AI inhibits aromatase, an enzyme that transforms adrenal-cortex androgens into estrogen, thereby suppressing endogenous estrogen production [13–15]. Another therapeutical option is surgery. As rare cases of malignant transformation have been reported, cytoreduction surgery with debulking have also been suggested for women who have completed their family. In our case, the patient was young, desired pregnancy and presented a limited extension of DPL, so only tumor excision was performed.

To our knowledge, no previous study has documented DPL histopathologic modifications after AI treatment. In our case, we found edema and fibrosis after 6 months of treatment with AI and GnRH agonist. These phenomena could explain the decrease in volume.

## CONCLUSION

Medical treatments are the first line since they allow local disease to be controlled, thus facilitating cytoreduction surgery. The association of AI with GnRH agonist seems to be the best option.

## CONFLICT OF INTEREST STATEMENT

None declared.

## FUNDING

The authors have not declared a specific grant for this research from any funding agency in the public, commercial, or not-for- profit sectors.
